# Graphene/Carbon Paper Combined with Redox Active Electrolyte for Supercapacitors with High Performance

**DOI:** 10.3390/polym11081355

**Published:** 2019-08-16

**Authors:** Yanlin Xia, Youtian Mo, Wei Meng, Xusheng Du, Chuanguo Ma

**Affiliations:** 1 Institute of Advanced Wear & Corrosion Resistance and Functional Materials, Jinan University, Guangzhou 510632, China; 2 Guangxi Key Laboratory of Information Materials, Guilin University of Electronic Technology, Guilin 541004, China

**Keywords:** cellulose, carbon paper, graphene, redox active electrolyte, supercapacitor

## Abstract

Graphene/carbon paper is prepared by pyrolyzing graphene modified cellulose filter paper and directly used as a binder-free electrode to assemble a supercapacitor (SC) with a redox active electrolyte, containing a Fe^3+^/Fe^2+^ additive. By the graphene incorporation and the carbonization of the cellulose fibers, both the microstructure and the electrical conductivity of the carbon paper are promoted greatly. The filter paper derived carbon (FPC) electrode exhibits a specific capacitance (*C*_s_) of 2832 F·g^−1^ in a 1 M H_2_SO_4_ + 0.5 M Fe^3+^/Fe^2+^ electrolyte at 1 A·g^−1^, which is about 81 times that in a normal H_2_SO_4_ electrolyte. With the modification of graphene, the capacitive performance of the SC is enhanced further and a remarkable *C*_s_ of 3396 F·g^−1^ at 1 A·g^−1^ is achieved for a graphene modified filter paper carbon (GFPC) electrode, which remains at ~632 F·g^−1^ at 10 A·g^−1^. The free standing GFPC electrode also exhibits good cycling stability (93.8% of capacitance retention after 2000 cycles) and an energy density of 118 Wh·kg^−1^ at a power density of 500.35 W·kg^−1^, all of which are much higher than those of FPC. These encouraging results suggest that the graphene modification of electrode materials combined with a Fe^3+^/Fe^2+^ redox active electrolyte is a prospective measure to fabricate SC with an ultrahigh performance.

## 1. Introduction

In recent years, clean and sustainable energy sources and new technologies related to energy conversion and storage have been attracting more and more attention [1]. Supercapacitors (SC) have the characteristics of fast charging and discharging speed, high power density, long life, environmental protection, and safety. In order to improve the performance of SC, different attempts have been made, including utilizing various electrolytes, developing novel electrode materials by controlling the nanostructure, preparing composite materials, or doping foreign heteroatoms [2,3,4,5,6].

Carbon-based electrode materials play an important role in the development of SC [7,8,9]. The increasing demand for high-performance SC has attracted a great deal of research into the top-level design and precise control of complex three-dimensional carbon structures [10,11]. Both the porous structure and surface area of the electrode materials can be optimized through reasonable design to improve their electrochemical properties. Cellulose, the most abundant organic resource on earth, is advantageous for the development of many devices because of its recyclability, abundance, and bio-compatibility [12,13]. Cellulose fibers exhibit a unique structure derived from their biological origin [14]. Moreover, they have some great properties, such as biocompatibility, flexibility, transparency, high mechanical strength, and biodegradability. The discovery of cellulose as a smart material has made it more favorable for the development of flexible and renewable sensors, actuators, and printed electronics [15,16]. One important type of filter paper (FP) is made of natural cellulose fibers and is widely used in various laboratories. There are numerous pores in FP through which liquids can pass. Therefore, carbonized FP can reserve the porous structure of the cellulose fibers and be a good candidate for electrodes in energy conversion and storage devices. As one type of advanced carbonaceous materials, two-dimensional graphene sheets exhibit excellent physical and chemical properties, high theoretical surface area, and excellent theoretical specific volume. Such sheets can be chemically functionalized, dispersed in polymer matrices, and deoxygenated to yield novel composites [17,18,19]. Flexible macroscopic assemblies of graphene, graphene oxide, or related composites have been used as electrodes for flexible SC [20].

Redox active electrolytes have been recently demonstrated to be an effective method to improve the electrochemical properties of SC [5,21,22]. They are normally prepared by adding redox additives into the electrolyte. Roldan et al. reported an increase in the specific capacitance (*C*_s_) of active carbon from 320 to 901 F·g^−1^ by adding hydroquinone to a H_2_SO_4_ electrolyte [22]. In another work, potassium iodide was also used as redox additive in the electrolyte, and the *C*_s_ of the biomass-derived carbon electrode in the resulting active electrolyte was tested to be 912 F·g^−1^, which is much higher than that of 472 F·g^−1^ in the normal electrolyte without the additive [23]. Such improvement in capacitance was attributed to the additional faradaic reactions of the additive at the electrode/electrolyte interface. Other kinds of redox active electrolytes, such as those containing Fe(CN)_6_^3−^ [24], VOSO_4_ [25], p-hydroxyaniline [26], and Fe^3+^/Fe^2+^ [27,28], were also prepared and investigated. Of these, active electrolytes containing a Fe^3+^/Fe^2+^ redox couple are easily available and cost-saving, and have been demonstrated as promising electrolytes in SC, where polyaniline (PANI) exhibits an enhanced *C*_s_ of 1062 F·g^−1^ at a current density of 2 A·g^−1^, nearly 2-fold that in normal electrolytes [28].

Although various carbon electrodes and active electrolytes have been developed for capacitors, little information on the capacitive performance of graphene modified carbon paper in Fe^3+^/Fe^2+^ active electrolytes is available so far. In this work, a graphene modified filter paper derived carbon (GFPC) paper will be fabricated by the incorporation of graphene sheets into the cellulose filter paper and the following pyrolysis at high temperature. The resulting carbon paper is used directly as free-standing electrode in the SC, with a redox active electrolyte containing Fe^3+^ and Fe^2+^ ions. The effect of the graphene modification and the usage of redox additive electrolyte on the electrochemical capacitive performance of the assembled SC will be investigated in detail.

## 2. Experimental 

### 2.1. Preparation of Filter Paper-Derived Carbon (FPC) Electrode and Graphene Modified Filter Paper-Derived Carbon (GFPC)

Filter paper (FP, Hangzhou special paper Co., LTD., Hangzhou, China) was cut into pieces with an area of 1 × 1 cm^2^. They were put into graphene aqueous slurry (5%; number of graphene layers: <10 layers; average particle size: 0.5–3 μm; Chengdu Organic Chemicals Co., Ltd., Chengdu, China) and subjected to ultrasonic irradiation for 30 min. After being dried at 100 °C for 8 h, GFP was obtained. Both FP and GFP were pyrolyzed in a tubular furnace at a constant heating rate of 5 °C·min^−1^ from room temperature to 300 °C, and maintained at 300 °C for 30 min under N_2_ flow, then heated at a constant rate of 5 °C·min^−1^ from 300 to 800 °C, and maintained at 800 °C for 1 h. Finally, FPC and GFPC were obtained as freestanding carbon papers. Ten samples were measured for each paper material, and the weight of the paper before and after the graphene modification was recorded to calculate the weight change with a precise analytical balance.

### 2.2. Characterization

X-ray diffraction (XRD) patterns were collected using an X-ray diffractometer (UItima IV), (Rigaku Corporation, The Woodlands, TX, USA), based on Cu-Ka radiation, with a scanning angle from 0° to 60° at a rate of 5°·min^−1^. Fourier-transform infrared (FTIR) and Raman spectra were taken on a Fourier-transform infrared spectrometer (iS50, EQUINOX 55, VERTEX70, karlsruhe, France) and Raman spectrometer (LabRAM HR Evolution, HORIBA, Kyoto, Japan), respectively. The FTIR wavenumber range was 500–4000 cm^−1^. The Raman wavenumber range was 450–3000 cm^−1^, and the laser power used was set to be 532 nm. The scanning electron microscopy (SEM) images were obtained on a Phenom XL instrument (Phenom XL instrument, Shanghai, China). The thermogravimetry analysis (TG) (TGA/DSC 3+ instrument, Mettler, Zürich, Switzerland) curve was recorded from 40 to 900 °C at a ramping rate of 20 °C·min^−1^ under an Ar atmosphere. The electrochemical measurements were conducted by an electrochemical working station (CHI 760e) (CH Instruments, Inc., Austin, TX, USA). The resistance of the samples was tested using a KDB-1 digital four-probe resistance tester (Guangzhou Kunde Technology Companies, Guangzhou, China). The ionic conductivity of different electrolyte solutions was measured by a DDS-11A conductivity meter (Shanghai Zhiguang instrument Co., Ltd., Shanghai, China).

### 2.3. Electrochemical Measurement

**Figure 1:**
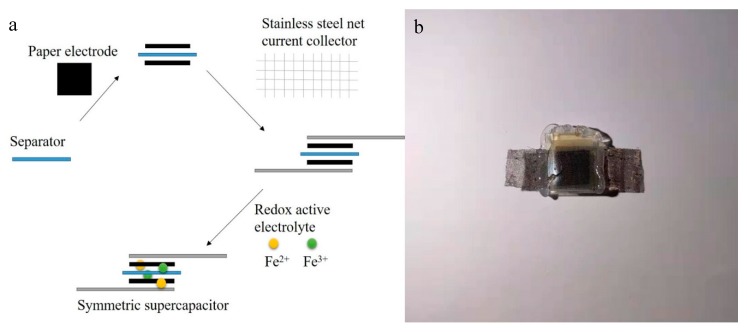
The assembly step (**a**) and a digital photo image of the positive view (**b**) of the supercapacitor.

The electrochemical performance of the obtained electrodes was characterized by cyclic voltammetry (CV), galvanostatic charge–discharge (GCD), and electrochemical impedance spectroscopy (EIS) in a two-electrode system. The symmetrical SC was configured with FPC or GFPC as the electrode, porous plastic film (PP/PE, EVOH and Nylon film, Mitsubishi, Xian, China) as the separator, and stainless steel net as current collector, as shown in Figure 1. A certain amount of electrolyte was added to the separator sandwiched in the two paper electrodes with a dropper and the assembled capacitor was wrapped with PE plastic film and sealed with RTV silicone adhesive/sealant to avoid leakage from the electrolyte. The electrochemical tests of the symmetric SC were performed using 1 M H_2_SO_4_ with different concentrations of Fe^3+^/Fe^2+^ as electrolytes. The total capacitance (*C*_total_) of the SC was calculated from the galvanostatic discharge process, according to the following Equation (1):

*C*_total_ = (*I* × *t*)/(*V* × *m*) (1)

where *I* is the discharge current (A), *t* is the discharge time (s), *V* is the voltage change (V), and *m* is the total mass of the active material for both electrodes (g). 

The specific capacitance of the single electrode was thus calculated as Equation (2):

*C*_s_ = 4 × *C*_total_ (2)

The energy density (*E*) and the power density (*P*) were calculated based on the following Equations (3) and (4):

*E* = (*C*_total_ × *V*^2^)/(2 × 3.6) (3)

*P* = *E*/*t* (4)

where *C*_total_ is the total capacitance of two-electrode cell (F·g^−1^), *V* is the voltage of the supercapacitor (V), and *t* is the discharge time (s) [28].

## 3. Results and Discussion

**Figure 2:**
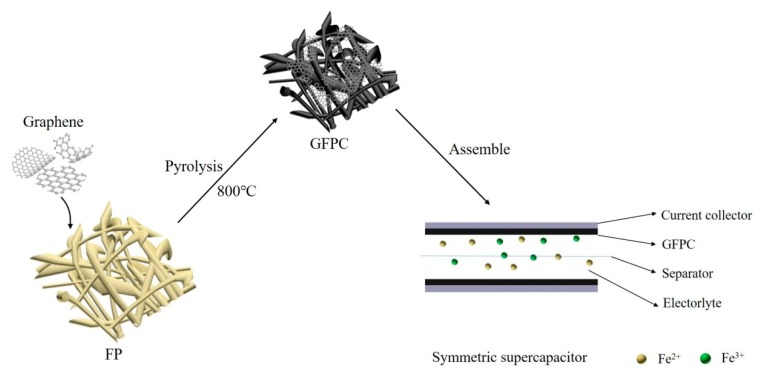
Illustrative schematic of the fabrication of the graphene modified carbon electrode and supercapacitors.

**Figure 3:**
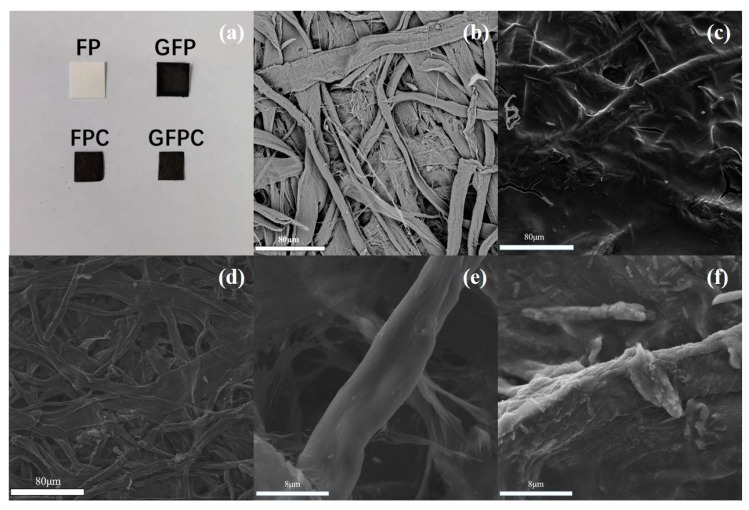
Digital photo image (**a**) and scanning electron microscopy (SEM) images of (**b**) FP, (**c**) GFP, (**d**,**e**) FPC, and (**f**) GFPC.

Cellulose filter paper was used as the polymer precursor for the carbon paper electrode. The schematic of the fabrication of the electrode and the corresponding SC is illustrated in Figure 2. Before carbonization or the modification with graphene, FP was white and clean, as shown in Figure 3a. It can be found that FP is mainly composed of crisscrossed fibers (Figure 3b). With the incorporation of graphene, the resulting GFP became black, as shown in Figure 3a, implying the successful modification of FP with graphene after the ultrasonic treatment of FP in the graphene slurry. In its SEM image, it can be also seen that the cellulose fiber surface was covered by graphene sheets (Figure 3c). After the pyrolysis of cellulose fibers, both FPC and GFPC showed a black color, and the freestanding paper form was reserved (Figure 3a). However, their microstructure was different. As shown in Figure 3d, FPC exhibited a nonwoven fiber structure, where the carbon fiber surface was relatively clean. In contrast, the fiber surface was much rougher in the SEM image of GFPC (Figure 3f), which could be due to the scrolling and folding of graphene sheets deposited on the carbon fiber surface.

**Figure 4:**
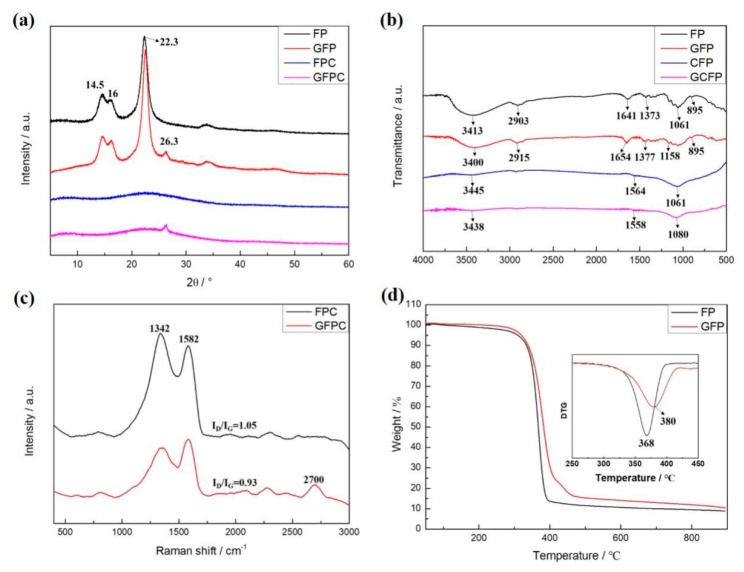
(**a**) XRD pattern, (**b**) FT-IR pattern, (**c**) Raman spectra, (**d**) TGA of FP and GFP. Inset in (**d**) is DTG of FP and GFP.

The XRD patterns of FP and GFP are shown in Figure 4a. Three typical peaks appeared at 2θ of 14.5°, 16° and 22.3°, corresponding to the diffraction (010), (001), and (011) planes of cellulose, respectively [29,30]. The pyrolysis of cellulose resulted in a broad peak at 2θ of 23° in the XRD patter of FPC, which can be assigned to the (002) plane of carbon materials from the carbonization of cellulose fibers in FP. The peak at 2θ of 26.3° in the XRD patterns of GFPC and GFP can be indexed to be the (002) plane of graphene [31], indicating the successful modification of graphene on FP, and the crystallinity of graphene changed little after pyrolysis. 

As shown in Figure 4b, the characteristic absorption peaks of FPC at 3413 and 1641 cm^−1^ are assigned to O–H stretching in hydroxyl groups and O–H bending, whereas the bands at and 1373 cm^−1^ are associated with the C–H stretching and C–H bending, respectively. C–O anti-symmetric stretching and C–O–C stretching appeared at 1159 and 895 cm^−1^, respectively [32,33]. After the carbonization, new peaks appeared at 1564 cm^−1^, and can be attributed to the stretching vibrations of C=C, indicating that an aromatic structure was generated by the carbonization. In addition, the presence of the peaks of 3445 and 1061 cm^−1^ indicates that the carbonized material contained hydroxyl or carboxyl functional groups.

The Raman spectrum of GFPC displayed three prominent peaks, namely, the G peak around 1582 cm^−1^, D peak around 1342 cm^−1^, and 2D peak at 2700 cm^−1^, as shown in Figure 4c. The formation of a G band is due to the in-plane bond stretching of sp^2^ carbon in graphene [34]. The D band depends strongly on the amount of disorder in the graphitic material [35]. The graphitic degree of carbon nanomaterials can be illustrated via the intensity ratio value of the D band to the G band (*I*_D_/*I*_G_) [36]. The *I*_D_/*I*_G_ for FPC was 1.05, which is little higher than that of GFPC (0.93), confirming again the presence of graphene sheets in GFPC.

TGA was carried out to study the thermal stability of the FP and GFP. As shown in Figure 4d, both FP and GFP experienced a rapid decomposition, starting from 250 to 400 °C. The strong peaks centered at 350–400 °C in the corresponding DTG curves are attributed to the pyrolysis of cellulose in the samples [37]. The DTG curves suggest that FP reached the maximum degradation rate at 368 °C, which is much less than that for the GFP (380 °C), illustrating that the presence of graphene sheets covering cellulose fibers promotes the thermal stability of the polymer fibers.

**Figure 5:**
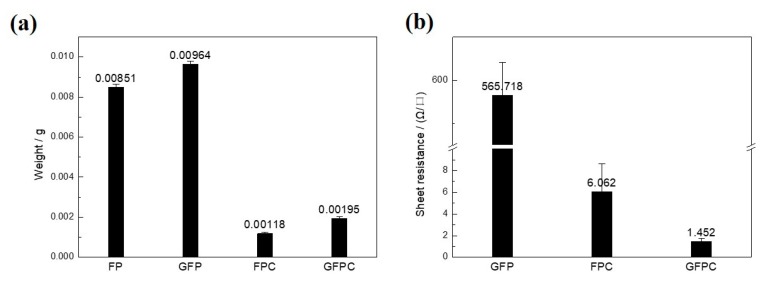
(**a**) The average weight of materials of FP, GFP, FPC, and GFPC, (**b**) the sheet resistance of GFP, FPC, and GFPC.

A histogram of the average weight and sheet resistance of FP and its derived materials is shown in Figure 5. The graphene content in the conductive papers can be obtained by the measurement of the weight change before and after the graphene modification. As shown in Figure 5a, after the ultrasonic treatment of the filter paper in graphene slurry, 11.7 (± 0.6) wt % graphene of the resulted GFP was loaded and deposited onto the surface of the cellulose fibers in FP. The graphene content increased to be ~39.5 wt % in GFPC, which could be due to the low yield of the carbonization of cellulose fibers. The graphene sheets introduced in the filter paper also affected the electrical conductivity of the GFP and GFPC. FP is electric insulated and its resistance is too large to be detected by the four-probe electrical resistance tester used in this work. After the pyrolysis, FPC showed a sheet resistance of 6.062 Ω/□ (Figure 5b). With the modification of graphene and carbonization of GFP, an even lower resistance of 1.452 Ω/□ for GFPC was achieved, confirming the improved electrical conductivity with the incorporation of graphene in the carbon paper. This means that it is a better electrode in SCs than FPC.

**Figure 6:**
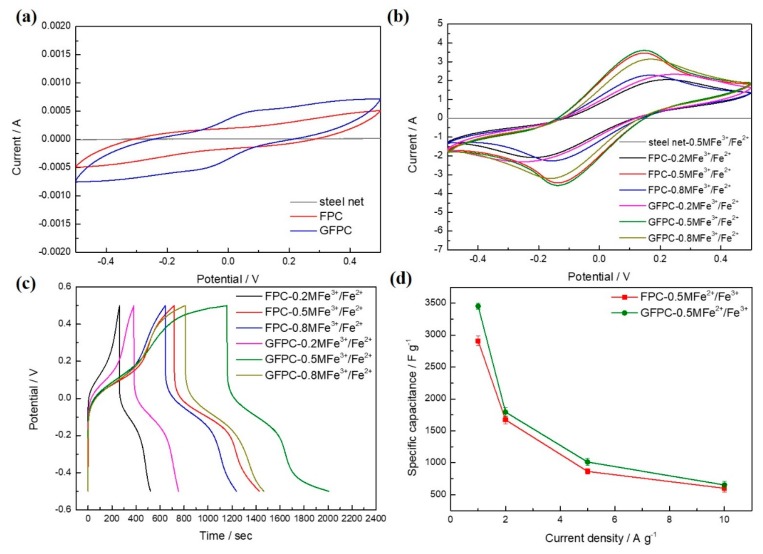
(**a**) The cyclic voltammetry (CV) curves of the FPC and GFPC in 1 M H_2_SO_4_ at a scan rate of 5 mV·s^−1^, (**b**) the CV curves of the FPC and GFPC in 1 M H_2_SO_4_ + 0.2/0.5/0.8 M Fe^3+^/Fe^2+^ electrolytes at a scan rate of 5 mV·s^−1^, (**c**) the galvanostatic charge–discharge (GCD) curves of the FPC and GFPC in 1 M H_2_SO_4_ + 0.2/0.5/0.8 M Fe^3+^/Fe^2+^ electrolyte of 1 A·g^−1^, (**d**) the specific capacitance of FPC and GFPC with different current densities in a 1 M H_2_SO_4_ + 0.5 M Fe^3+^/Fe^2+^ electrolyte.

The electrochemical performances of the filter paper-derived carbon electrode were investigated by using CV and GCD measurements within the potential window of −0.5 to 0.5 V, as shown in Figure 6. The CV curves of the FPC and GFPC electrodes and steel net at a scan rate of 5 mV·s^−1^ in a 1 M H_2_SO_4_ electrolyte are shown in Figure 6a. Stainless steel net was used as the current collector in SCs. It is easily available and cheaper than other current collectors, such as carbon mate. As it is possible for the current collector to have contact with the active electrolyte, its electrochemical activity has to be investigated to see its contribution to the capacitive performance of the SC. Interestingly, different from the high activity of the carbon materials, stainless steel net does not show any significant electrochemical activity to the redox reaction of the redox additive in the electrolyte in the same potential window, and its CV curve is almost close to a straight line, indicating its negligible contribution to the *C*_s_ of the whole electrodes. The poor electrochemical activity of the stainless steel to the redox active electrolyte could be likely due to its specific chemical composition, such as element Cr, Si, P, and Mn, making it inert to the redox reaction of Fe^3+^/Fe^2+^. However, this means it is very stable in contact with the redox active electrolyte and could be a stable current collector in the capacitors. The current density of GFPC is obviously larger than that of FPC, confirming that the modification of the carbon paper electrode with graphene sheets improves its electrochemical performance. Figure 6b shows the CV curves of the FPC or GFPC in redox active electrolyte at the same scan rate. Compared with the CV curves of the symmetric SC with a 1 M H_2_SO_4_ electrolyte (Figure 6a), a pair of strong peaks centered at −0.15 and 0.15 V can be observed for the SC with 1 M H_2_SO_4_ + Fe^3+^/Fe^2+^ electrolytes, suggesting a significant contribution of the Fe^3+^/Fe^2+^ redox couple in the active electrolyte [28]. In addition, it can be found that the area surrounded by CV curves of the GFPC electrode is obviously larger than that of the FPC electrode in the same electrolyte, confirming again the positive effect of the modification of the carbon paper with graphene on its electrochemical property in the redox active electrolyte. The GCD curves for the SC with a redox active electrolyte containing different concentrations of Fe^3+^/Fe^2+^ at a current density of 1 A·g^−1^ are shown in Figure 6c. High voltage drops can be seen in all the GCD curves, which could be due to the larger inner resistance of the SC, possibly originating from the contact resistance between the steel mesh, the conductivity of carbon electrodes themselves, and polarization of the electrochemical process of the electrode. This means a SC with an even better performance could be obtained by a further decrease of such resistances. The calculated *C*_s_ of FPC electrode was 1030 F·g^−1^ in 1 M H_2_SO_4_ + 0.2 M Fe^3+^/Fe^2+^, 2832 F·g^−1^ in 1 M H_2_SO_4_ + 0.5 M Fe^3+^/Fe^2+^, and 2372 F·g^−1^ in 1 M H_2_SO_4_ + 0.8 M Fe^3+^/Fe^2+^, respectively. The *C*_s_ of the electrode did not increase monotonously with the increase in Fe^3+^/Fe^2+^ concentration, and the optimum concentration of the Fe^3+^/Fe^2+^ redox couple was 0.5 M. In the case of KI as the redox additive in the electrolyte, the decrease in the capacitance at even higher concentrations was attributed to the aggregation of free ions and the crystallization of KI in the gel electrolyte [38]. However, this is not the case here, as the conductivity of all three electrolytes with different Fe^3+^/Fe^2+^ concentrations was tested to be the same, i.e., 24.4 mS/cm. Therefore, the reason for the decrease in the capacitance of the electrolyte of 1 M H_2_SO_4_ + 0.8 M Fe^3+^/Fe^2+^ is not clear at present, and further study on this is underway. With the modification of graphene on the electrode, the *C*_s_ of the carbon paper increased, to 1488 F·g^−1^ (GFPC-0.2 M Fe^3+^/Fe^2+^), 3396 F·g^−1^ (GFPC-0.5 M Fe^3+^/Fe^2+^), and 2628 F·g^−1^ (GFPC-0.8 M Fe^3+^/Fe^2+^). Therefore, among all the symmetric SCs, the GFPC electrode in 0.5 M Fe^3+^/Fe^2+^ exhibited the highest *C*_s_ at the same current density, confirming it is the optimized configuration for the SC. This is different from a previous report on polyaniline-based SC, where 0.8 M was the best [28]. This could be due to the different electrode used in the SC. Moreover, with the increase in GCD current density, the *C*_s_ of the GFPC electrode decreased, which could be due to the inadequate time for active species in electrolyte diffusion into the inner pores at higher current density [38,39,40]. Such a remarkable *C*_s_ at certain current density could be one of the highest *C*_s_ among those studies on carbon paper based electrodes (Table 1), indicating the significant contribution of the redox active additive in the electrolyte system.

**Figure 7:**
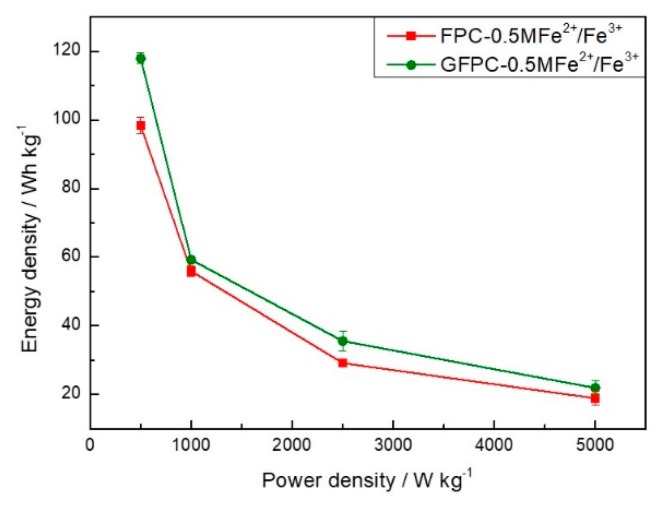
Ragone plots of FPC and GFPC symmetric SCs in a 1 M H_2_SO_4_ + 0.5 M Fe^3+^/Fe^2+^ electrolyte.

The energy density of the energy storage device is a very important factor. The symmetric SC assembled by GFPC and a 1 M H_2_SO_4_ + 0.5 M Fe^2+^/Fe^3+^ electrolyte delivered an energy density as high as 118.00 Wh·kg^−1^ at a power density of 500.35 W·kg^−1^. As shown in Figure 7, it is also a little larger than that of the FPC-based SC with the same redox active electrolyte (for instance, 98.33 Wh·kg^−1^ at a power density of 500.35 W·kg^−1^), indicating the advantage of the graphene modification of the carbon paper electrode.

**Figure 8:**
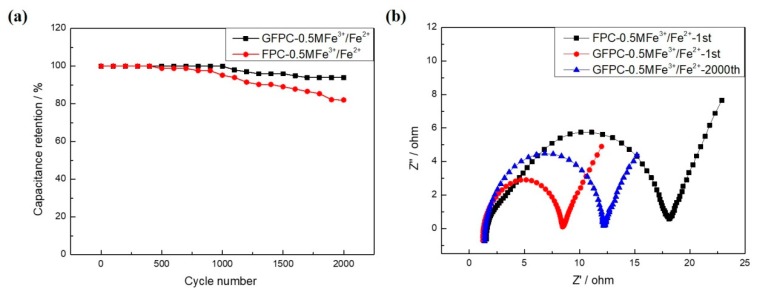
(**a**) Cycling performance of GFPC in a 1 M H_2_SO_4_ + 0.5 M Fe^3+^/Fe^2+^ electrolyte of 10 A·g^−1^, (**b**) Nyquist plots of symmetric FPC and GFPC supercapacitors in 1 M H_2_SO_4_ + 0.5 M Fe^3+^/Fe^2+^ electrolytes, measured at a frequency range of 100 kHz to 0.01 Hz.

The cyclic stability of the FPC and GFPC electrode in the redox electrolyte was also studied. As shown in Figure 8a, the capacitance of GFPC remained at 93.8% after GCD for 2000 cycles, which is higher than that of FPC (82.4%). This reveals that during successive charge–discharge cycling, the GFPC exhibited not only better rate capability (Figure 6d), but also higher cyclic stability than FPC, indicating graphene in the carbon paper electrodes promotes the long-term stability of the SC with the highly active electrolyte. 

EIS was measured to further understand the electrochemical behaviors of the SC. Figure 8b shows the Nyquist plots of FPC and GFPC in a 1 M H_2_SO_4_ + 0.5 M Fe^3+^/Fe^2+^ electrolyte. It can be seen that both plots of FPC and GFPC exhibit two distinct traits: a semicircle in the high frequency range and a sloped line in the low frequency range. The diameter of the semicircle in the high frequency region represents the charge-transfer resistance (*R*_ct_) at the interface between the electrolyte and electrode. The *R*_ct_ of GFPC was clearly much less than that of FPC, as shown in Figure 8b. The *R*_s_ can be obtained from the x-intercept in high frequency, which is associated with the sum of the intrinsic resistance of the active material, electrolyte solution resistance, and the contact resistance at the electrode-electrolyte interface [46]. GFPC showed a decreased *R*_s_ value of 1.28 Ω as compared with 1.61 Ω for FPC, which can be ascribed to the better electrical conductivity of GFPC than FPC. Moreover, the *R*_s_ of GFPC increased to be 1.43 Ω after 2000 cycles. The increase of the ohmic inner resistance of the SC could be responsible for the drop in the *C*_s_ after the multi-cycles [38].

## 4. Conclusions

In summary, freestanding carbon paper electrodes were prepared by the carbonization of cellulose filter paper at a high temperature. The incorporation of graphene in the paper improved the electrical conductivity and capacitive performance of the carbon paper electrodes. The redox active electrolyte containing a Fe^3+^/Fe^2+^ redox couple was combined with the carbon paper electrodes to assemble the symmetric SC, and the optimized concentration of the redox couple additive in the electrolyte was found to be 0.5 M. The GFPC electrode in the SC displayed a *C*_s_ as high as 3396 F·g^−1^ at 1 A·g^−1^, a good cyclic stability (93.8% capacitance retention after 2000 cycles), and a high energy density of 118 Wh·kg^−1^ at a power density of 500.35 W·kg^−1^, all of which was superior to that of the FPC electrode. This work demonstrated that the modification of a carbon paper electrode with graphene sheets, simultaneously combined with the use of a Fe^3+^/Fe^2+^ redox active electrolyte could be an effective approach to prepare supercapacitors with an ultrahigh performance.

## Figures and Tables

**Table 1:** Comparison of specific capacitance (*C*_s_) of carbon based electrodes in redox active electrolyte.

Electrode Materials	Redox Active Electrolyte	*C*_s_ (F·g^−1^)	Reference
Active carbon (from mango kernels)	1 M H_2_SO_4_ + 50 mM p-hydroxyaniline (PHA)	587.1 F·g^−1^ at 1 A·g^−1^	[26]
Active carbon	H_2_SO_4_ + p-benzenediol	474.29 F·g^−1^ at 0.83 A·g^−1^	[41]
Active carbon	1 M H_2_SO_4_ + 0.38 M hydroquinone	901 F·g^−1^ at 2.65 mA·cm^−1^	[22]
Nitrogen-doped porous carbon	1 M H_2_SO_4_ + 0.08 M NaMoO_4_	1416F·g^−1^ at 15 A·g^−1^	[42]
MWCNTs/PANI	1 M H_2_SO_4_ + 0.05 M KI	3331 F·g^−1^ at 1 A·g^−1^	[43]
Active carbon	1 M H_2_SO_4_ + 0.05 M FeBr_3_	885 F·g^−1^ at 2 A·g^−1^	[44]
Porous carbon microspheres	1 M HNO_3_ + 0.12 M CuCl_2_	4700 F·g^−1^ at 5 mV·s^−1^	[45]
CNTs/PANI	1 M H_2_SO_4_ + 0.02 M Fe^3+^/Fe^2+^	1128 F·g^−1^ at 5 A·g^−1^	[27]
Graphene/Carbon (from filter paper)	1 M H_2_SO_4_ + 0.5 M Fe^3+^/Fe^2+^	3396 F·g^−1^ at 1 A·g^−1^	This work
